# An Artificial Intelligence Characterised Functional Ingredient, Derived from Rice, Inhibits TNF-α and Significantly Improves Physical Strength in an Inflammaging Population

**DOI:** 10.3390/foods9091147

**Published:** 2020-08-20

**Authors:** Kathy Kennedy, Brian Keogh, Cyril Lopez, Alessandro Adelfio, Brendan Molloy, Alish Kerr, Audrey M. Wall, Gaël Jalowicki, Thérèse A. Holton, Nora Khaldi

**Affiliations:** Nuritas Ltd., D02 RY95 Dublin, Ireland; kennedy.kathy@nuritas.com (K.K.); Keogh.brian@nuritas.com (B.K.); cyrlop06@hotmail.com (C.L.); Adelfio.Alessandro@Nuritas.com (A.A.); molly.brendan@nuritas.com (B.M.); kerr.alish@Nuritas.com (A.K.); Jalowicki.gael@nuritas.com (G.J.); holton.therese@nuritas.com (T.A.H.); Nora@Nuritas.com (N.K.)

**Keywords:** inflammation, bioactive peptides, tumor necrosis factor alpha, artificial intelligence, functional ingredient, inflammaging

## Abstract

Food-derived bioactive peptides offer great potential for the treatment and maintenance of various health conditions, including chronic inflammation. Using in vitro testing in human macrophages, a rice derived functional ingredient natural peptide network (NPN) significantly reduced Tumour Necrosis Factor (TNF)-α secretion in response to lipopolysaccharides (LPS). Using artificial intelligence (AI) to characterize rice NPNs lead to the identification of seven potentially active peptides, the presence of which was confirmed by liquid chromatography tandem mass spectrometry (LC-MS/MS). Characterization of this network revealed the constituent peptides displayed anti-inflammatory properties as predicted in vitro. The rice NPN was then tested in an elderly “inflammaging” population with a view to subjectively assess symptoms of digestive discomfort through a questionnaire. While the primary subjective endpoint was not achieved, analysis of objectively measured physiological and physical secondary readouts showed clear significant benefits on the ability to carry out physical challenges such as a chair stand test that correlated with a decrease in blood circulating TNF-α. Importantly, the changes observed were without additional exercise or specific dietary alterations. Further health benefits were reported such as significant improvement in glucose control, a decrease in serum LDL concentration, and an increase in HDL concentration; however, this was compliance dependent. Here we provide in vitro and human efficacy data for a safe immunomodulatory functional ingredient characterized by AI.

## 1. Introduction

The severe impact that chronic, low grade inflammation exerts on human health has been decidedly revealed over the past number of decades [1], emerging as the principal causative factor underlying most chronic diseases [2]. While acute inflammatory responses are vital for the resolution of infection or physical trauma [3], failure to appropriately resolve inflammation ultimately results in tissue damage, leading to conditions such as psoriasis, arthritis and colitis [4], increased risk of developing metabolic diseases [1,5], and, indeed, cancer [6]. Chronic dysregulation of inflammatory responses results in a systemic pro-inflammatory state, typified by elevated levels of cytokines such as interleukin (IL)-1, IL-6, IL-8 and tumor necrosis factor alpha (TNF-α) [7]. Implicit in many of these disorders is the fact that chronic low-grade inflammation is characteristically experienced during ageing, in a phenomenon known as “inflammaging” [7,8]. Inflammaging increases the risk of pathologic conditions and age-related diseases, and it has also been associated with increased skeletal muscle wasting, strength loss, and functional impairments [9,10]. Inflammaging is characterized by typically a 2−4-fold increase in levels of pro-inflammatory cytokines such as TNF-α [11] with values in healthy young populations averaging 3.21 (±4.04) pg/mL [12].

TNF-α is a pleiotropic cytokine, which has been identified as a central mediator of chronic inflammatory responses in many diseases [13]. Signaling through two distinct, membrane expressed receptors, TNFR1 and TNFR2, TNF-α modulates important cellular functions such as proliferation, differentiation, and apoptosis [14,15]. Crucially, TNF-α is not only responsible for mediating inflammatory effects directly, but also through the induction of a pro- inflammatory cytokine cascade [16], earning it the reputation as a “master regulator” of inflammation [14]. Accordingly, targeting TNF-α has emerged as an important strategy for the treatment of chronic inflammatory diseases [17,18]. While undoubtedly a landmark development in terms of patient health, anti-TNF drugs present known limitations in terms of safety, potentially leading to increased bacterial infections [18].

Due to the functional diversity associated with endogenous peptides, many are investigating the potential of exogenous bioactive peptides from food and plant sources for the prevention and treatment of various conditions [19], including inflammation [20,21]. Distinct advantages of peptide-based approaches include pleiotropic functionality, high selectivity and efficacy, as well as favorable safety and tolerability profiles [22]. However, the functional food-based approach has benefitted from the recent advancements of various “omics” techniques. The use of bioinformatics has proven influential in the discovery of bioactive peptides from natural sources [23,24,25], with health benefits being associated with the presence of multiple bioactive peptides in a single natural peptide network (NPN)/hydrolysate discovered through artificial intelligence (AI) [26]. While there is an opportunity for functional food in immunomodulatory preventative strategies [27,28], there has been limited clinical data to suggest that functional foods can promote good health [29]. Accordingly, there is a need for a characterized and scientifically validated functional ingredient which can attenuate overproduction of pro-inflammatory cytokines and an AI approach is increasingly seen as an opportunity to overcome these issues by deciphering large, complex datasets, such as functional food proteomes [26,30,31].

In order to functionally assess the effects of interventions designed to counteract the development of frailty and its associated state of inflammation, standardized, relevant testing methods are required. These standardized tests include the short physical performance battery (SPPB) which comprise of walking speed of a person over a distance of 4 meters, the ability to stand unassisted (standing balance), the strength of hand grip in both arms, and the ability to stand up from a chair five times without using their arms (repeated chair raise test). All tests have been extensively validated [32]. In subjects >65 years of age, the SPPB is an effective and inexpensive protocol that can be carried out with minimal equipment. It has been shown to consistently score high for reliability, validity, and responsiveness [33]. It is important to note that hand grip strength does not provide a valid means to evaluate the efficacy of invention therapies to increase muscle function in an older population and as such, is likely to be less sensitive to interventions than lower limb tests, such as the chair sit test. These tests can be useful as predictors of the likelihood of events such as falling. For example, the most strongly associated with falling in woman was gait speed whereas in men, it was the chair test [34]. It may be possible to affect these readouts with daily exercise and a significant increase in protein intake. However, in this study, we did not alter daily activity nor protein intake, indicating that the NPN is acting independent of any lifestyle changes. Apart from its role in the development of frailty [35], TNF-α changes have been linked to key physiological processes that are associated with the development of diseases. Insulin signal transduction can be disrupted, resulting in insulin resistance [1], and subsequent development of Type 2 diabetes. This can also lead to thickening of blood vessels and compromised endothelial barrier strength and thus increases the risk of cardiovascular disease [8]. A state of chronic inflammation has also been associated with the development of atherosclerosis [36]. We thus incorporated measurements of circulating cytokines, HDL and LDL concentrations as well as glucose testing into our trial design to assess the effect of rice NPN on these parameters.

In this study, we use AI to identify and validate a natural peptide network (NPN), derived from rice, demonstrating anti-inflammatory activity in vitro. Using a machine learning approach, we further characterize rice NPN by identifying a number of constituent bioactive peptides. Finally, to assess associated health benefits of attenuating circulating TNF-α concentrations in an “inflammaging” population, a pilot study was conducted with daily administration of rice NPN over 12 weeks. We show that rice NPN significantly attenuated TNF-α production within 4 weeks of administration. Furthermore, we show significant improvements on oral glucose tolerance testing and a decrease in serum LDL with a concomitant increase in HDL. Finally, in the pilot study, significant physical gains observed, for example, with the chair stand and short physical performance battery tests. In short, an anti-inflammatory rice NPN may have significant benefits in age-related inflammation.

## 2. Materials and Methods

### 2.1. Peptide Prediction for Bioactivity and Natural Source Identification

To develop a functional peptide hydrolysate that can reduce TNF-α, a predictive machine learning approach was first used to predict peptides with anti-inflammatory activities. To assess this activity, an untargeted approach was applied; structured and unstructured data sources including scientific literature, patents, and public databases were interrogated for anti-inflammatory peptides, with a focus on those with the ability to attenuate TNF-α secretion. For quality assurance, all data were manually curated. A neural network predictive architecture was trained in 10-fold cross validation, once a reliable non redundant dataset of labelled anti-inflammatory peptides was attained (~10^4^ data points). The best models for the validation sets were refined on a set of peptides specifically labelled for TNF-α inhibition. This resulted in a smaller set of peptides (~2 × 10^2^), which included a set of proprietary peptides that had been previously validated for anti-TNF-αactivity in-house. The ensemble of the 10 resulting models was used to predict an additional set of proprietary peptides with experimentally determined effect on TNF-α secretion, exhibiting an accuracy higher than 85%.

The resulting predictor was finally used to identify bioactive peptides in the proteomes of all the food sources available from our in-house repository. In order to do that, we fragmented the top 10 most abundant proteins of each source into peptides with lengths from 4 to 30 amino acids. Then we scored the resulting set of ~6 × 10^6^ peptides using the predictor. We found *Oryza sativa* as the food source containing the highest number of predicted anti-inflammatory peptides to be unlocked from its most abundant proteins.

From these predicted anti-inflammatory peptides, a final set of 8 highly ranked novel peptides were selected for further investigation. All bioactive peptides used in this study were produced by GenScript (Piscataway, NJ, USA), where peptide sequence and purity (95%–99%) were validated by HPLC–MS/MS.

### 2.2. Natural Peptide Network Production

Following the high number of peptides predicted to reduce the expression of TNF-α found in the *Oryza sativa L.* subsp. *japonica* proteome, methods similar to Rein et al., 2019, were used to obtain a hydrolysate, rice NPN, which contained these peptides, including the final set of 8 highly ranked peptides [26]. Briefly, we solubilized brown rice protein and sequentially hydrolyzed it with food grade serine protease, in a pH and temperature controlled aqueous solution. The solution was dispersed using a high shear mixer in reverse osmosis water and pH adjusted using sodium hydroxide or hydrochloric acid for a final value of pH 6. The hydrolysis reaction was terminated by heating the solution to 85 °C for 10 min. Immediately after, the solution was rapidly cooled using a plate cooler and stored overnight at 4 °C. The solution was spray dried at 180 °C using a multi-stage Anhydro Spray Dryer at a facility accredited to FSC2200.

The study product was manufactured at Teagasc, Moorepark, under HACCP Food Quality Standards. No chemical additives were used, and the product was free of sulfites, sulfates and aflatoxins. The source material was produced in an ISO 22000 and HAACP compliant facility.

### 2.3. Sample Preparation and Mass Spectrometry Analysis

Samples (100 μL) were desalted and concentrated using 30-kDa Spin-X UF centrifugal concentrators (Corning Inc., Lowell, MA, USA). Each flow-through was then acidified in formic acid and cleaned of contaminants by solid phase extraction (SPE) on an Empore 96-well disk plate with C18-SD sorbent (3M, St. Paul, MN, USA). Eluates were lyophilized and resuspended in 0.1% TFA for analysis.

Samples were analyzed by nano LC-MS/MS with a Waters NanoACQUITY HPLC system (Waters Corporation, Milford, MA, USA) interfaced to a ThermoFisher Q Exactive (ThermoFisher Scientific Inc., Canoga Park, CA, USA). Peptides were loaded on a trapping column and eluted over a 75 μm analytical column with a 1 h gradient at a flow rate of 350 nL min^−1^. Both columns were packed with Luna C18 resin (Phenomenex, Torrance, CA, USA). The mass spectrometer was operated in data-dependent mode, with MS and MS/MS performed in the Orbitrap at 70,000 FWHM and 17,500 FWHM resolution, respectively. From the MS scan, the fifteen most intense ions were selected for MS/MS.

To confirm the presence of predicted peptides in rice NPN, raw data from the Q Exactive was processed by MaxQuant version 1.5.3.17 (Martinsried, Germany) [37] with the Andromeda search engine [38]. MS/MS spectra were searched against the Uniprot *Oryza sativa L*. subsp. *japonica* database. All searches were carried out in unspecific digestion mode with a minimum peptide length of 5 amino acids and a fragment mass tolerance of 20 ppm. A false discovery rate (FDR) of 0.01 was selected for both peptides and proteins. Database searches were performed with no fixed modifications and with oxidation (M) as a variable modification.

### 2.4. Cell Culture, Differentiation of Monocytes and Inflammation ELISA Assays

Human monocytic leukemia (THP-1) cells (ECACC collection; Sigma-Aldrich, St Louis, MO, USA) were maintained in culture in Roswell Park Memorial Institute medium (RPMI 1640, Lonza, Basel, Switzerland) supplemented with 1% L-glutamine, 10% heat-inactivated FBS and 1% penicillin–streptomycin. Cells were kept viable at 37 °C and 5% CO_2_.

To differentiate into macrophages, THP-1 cells (1x10^6^ cells/mL) were seeded in 24 well plates and treated with 100 nM phorbol-12-myristate-13-acetate (PMA; Sigma-Aldrich, St Louis, MO, USA) for 72 h at 37 °C, 5% CO2. After incubation, non-attached cells were aspirated, and adherent cells were treated with peptides (0.5 µg/mL), peptide network (0.5 and 5 µg/mL) or remained untreated. Docosahexaenoic acid (DHA) at 32.8 µg/mL was used as a positive control [39]. Following incubation for 24 h, LPS from *Escherichia coli* O127:B8 (Sigma-Aldrich, St Louis, MO, USA) was added at 100 ng/mL for 24 h at 37 °C, 5% CO_2_. Cell supernatants were collected and TNF-α concentrations were determined by ELISA according to the manufacturer’s instructions (BioLegend, San Diego, CA, USA). Absorbance was measured at 450 nm with a microplate spectrophotometer (SpectraMax M3, Molecular Devices, Sunnyvale, CA, USA).

### 2.5. Human Randomised, Double Blinded, Parallel Group, Placebo Controlled Clinical Trial

#### 2.5.1. Participants and Study Design

Forty volunteers participated in a randomized, double-blind, placebo-controlled, parallel study carried out in accordance with the Declaration of Helsinki. Written, informed consent was obtained from all participants and ethical approval was granted by the clinical research ethics committee of the Cork Teaching Hospitals. The trial was carried out under the supervision of Prof. Fergus Shanahan and was carried out at Atlantia Food Clinical Trials sites at Blackrock and Mallow, County Cork, Ireland (trial protocol NCT04450979). Eligible participants were healthy males and females aged 65–75 years with a BMI < 30 kg/m^2^. Participants were excluded if they had a chronic or infectious disease, were taking anti-inflammatory medications or hormone replacement therapy, or had an allergy to components of the test products. Subject anthropometric data are shown in Supplemental Table S1. Volunteers were randomly assigned to either the treatment (rice NPN) or placebo (maltodextrin) group, with 75% of subjects (*n* = 30) receiving the treatment and 25% (*n* = 10) the placebo. Subjects were supplied with a four-week supply of rice NPN or placebo and fully instructed on how to reconstitute the product. Subjects were instructed to take one dose each morning and one dose each evening for the study duration. Each 10 g dose was supplied in a pre-weighed sachet that was mixed with 200 mL of water until the product was fully resuspended and then consumed immediately. This study is considered a pilot study, since to our knowledge, it is the first study to investigate the anti-inflammatory potential of a food-derived peptide ingredient on circulating cytokines over 12 weeks in combination with the monitoring of upper and lower body strength through tests such as the chair stand test. In line with previous pilot studies [40], in the absence of effect size information, this study will serve to aid in the design of future such studies.

Fasting blood samples were collected at baseline and on week 4, 8, and 12 of the study. Blood samples were centrifuged, and the resulting serum stored at −80 °C until analysis.

##### 2.5.1.1. Hand Grip Test

Upper body muscle strength was measured using a hand grip test at baseline, as well as weeks 4, 8, and 12. The hand grip test was carried out using a dynamometer and grip strength was recorded. In brief, handgrip strength test was performed with the subject seated in a firm and stable straight-back chair with the shoulder adducted and in a neutral position, the elbow flexed at 90°, and the lower arm and wrist in a neutral position. The arm was not supported by a table, arm rest, or pillows. The appropriate use of the dynamometer was demonstrated before the subject performed a test hand grip. The dominant hand was tested, and one test hand grip was measured followed by 3 independent hand grips at maximal effort held for at least 5 s. The highest measure of the 3 independent hand grips displayed by the dynamometer was recorded in kilograms.

##### 2.5.1.2. Repeated Chair Stand Test

Lower body muscle strength was measured using a chair stand test. The repeated chair stand test was carried out by counting the number of complete chair stands performed in a 30 s period. These tests were carried out at baseline, and weeks 4, 8, and 12. This test is described in detail in Section 2.5.1.3 as part of the SPPB carried out at baseline and week 12.

##### 2.5.1.3. Short Physical Performance Battery (SPPB)

A short physical performance battery (SPPB) test score was generated at baseline and week 12. The SPPB is a group of measures that combines the results of the gait speed, chair stand and balance tests [41]. It has been used as a predictive tool for possible disability and can aid in the monitoring of function in older people. The scores range from 0 (worst performance) to 12 (best performance). The SPPB has been shown to have predictive validity showing a gradient of risk for mortality, nursing home admission, and disability. For the Short Physical Performance Battery (SPPB) testing, a combination of balance tests, gait speeds, and strengths were assessed as follows. For the balance tests, the subject must be able to stand unassisted without the use of a cane or walker, although assistance could be provided to allow the subject to stand. If at any point, the subject felt it was unsafe to carry out the test, the test was skipped and recorded as such. All movements were first demonstrated. Firstly, subjects were asked to stand with their feet together for 10 s. If the subject successfully carried out the task a score of 1 was given, otherwise a score of 0 was given and the assessor moved on to the gait speed test. In the case of a successful test, a semi-tandem stand test was performed. As before, this was demonstrated first. Subjects were asked to stand with the side of the heel of one foot touching the big toe of the other foot for 10 s. If the subject successfully carried out the task a score of 1 was given, otherwise a score of 0 was given and the assessor moved on to the gait speed test. In the case of a successful test, a tandem stand test was performed. The subject was asked to stand with the heel of one foot in front of and touching the toes of the other foot for 10 s. If the subject successfully carried out the task a score of 2 was given, if the subject held the stance for 3–9.99 s, a score of 1 was given, and for <3 s, a score of 0 was given. The sum of these scores was calculated for an overall score for each subject.

The gait speed test was used to assess how a subject normally walked over a four meter distance. A cane or walking aid was allowed if normally required. Subjects were instructed to walk at their normal pace and the test was demonstrated by the assessor. The assessor always walked beside the subject, but at the pace of the subject. The time taken to cover four-meters was recorded, from the time the subject entered the course until one foot completely exited. Scoring was assessed as follows; >8.70 s, 1 point; 6.21 to 8.70 s, 2 points; 4.82 to 6.20 s, 3 points; <4.82 s, 4 points.

In the SPPB, two different chair stand tests were used, the single and repeated chair tests. For the single chair test subjects were asked to stand up from a chair without using their arms. Again, the test was demonstrated by the assessor and the subject was asked if they felt safe to perform the test. Subjects were instructed to fold their arms across their chest and sit so that their feet were on the floor; then to stand up while keeping their arms folded across your chest. If the subject could not rise without using arms, it was assessed if they could rise from the chair using their arms. The result was recorded.

In the repeated chair stand test, subjects were asked if they thought it would be safe for them to try to stand up from a chair five times without using their arms. The correct procedure was demonstrated. Subjects were instructed to stand up as quickly as they could five times, without stopping in between. After standing up each time, they must completely sit down and then stand up again while keeping their arms folded across their chest. The time to complete this was recorded. The test was immediately stopped and recorded if the subject became tired or out of breath. The test was also stopped if the subject used their arms to rise from the chair, of if the test was not completed within 1 min.

Scoring in the SPPB was assessed as follows; if the subject used their arms to stand in the single test 0; if the subject stood without using their arms, they progressed to the repeated chair stand test, which was scored as follows; subject unable to complete 5 chair stands or completes stands in >60 s, 0 points; 16.70 s or more, 1 point; 13.70 to 16.69 s, 2 points; 11.20 to 13.69 s, 3 points; >11.19 s, 4 points. An overall SPPB score was obtained for all activities and recorded.

#### 2.5.2. Glucose Tolerance Test

Subjects fasted for at least ten hours prior to the visit, with only water allowed to be consumed within that period. A cannula was inserted into the subject’s non-dominant arm and an initial baseline 2 mL blood sample was collected. The subject drank 410 mL of Lucozade (70 kcal/100 mL, equivalent to 75 g of anhydrous glucose). The load was consumed within 5 min. The time to finish the load was recorded and additional blood samples were taken at 30, 60, 90, and 120-min post completion of the drink. This test was performed at baseline and week 12.

#### 2.5.3. Serum Cytokine Concentrations

Serum concentrations of TNF-α, IL-1β, IL-6 and C reactive protein (CRP) were measured using the Milliplex™ MAP Human Cytokine/Chemokine Magnetic Bead Panel (EMD Millipore Corporation, Billerica, MA, USA) according to the manufacturer’s instructions. Plates were read using the Luminex MAGPIX^®^ system and concentrations were calculated using xPONENT software (Luminex, Austin, TX, USA). HDL and LDL concentrations were also measured in the serum samples.

### 2.6. Statistics

For in vitro experiments, statistical analyses were performed using the statistical computing software R [42], significant differences from untreated controls were determined by one-way ANOVA followed by a Dunnett’s test. Data are presented as a percentage of untreated controls (mean ± SEM of at least 3 independent experiments).

For physicochemical analysis, following LC-MS/MS of rice NPN, all data were collected, and analyses carried out using in-house Python scripts. Graphs illustrating peptide physiochemical properties (amino acid length, overall charge, and percentage of hydrophobic residues) were created using the “ggplot2” R package.

For clinical data, mean change from baseline was determined for each time point and expressed as mean ± SEM for each treatment group. Total area under the curve (AUC) was calculated using the trapezoidal rule and treatment groups were compared by Student’s t-test. Additionally, for each time point, treatment groups were compared by ANOVA followed by Tukey’s Test. For all analyses, the *p* value < 0.05 was considered significant. Confounding factors, such as general compliance and compliance due to timing (i.e., overlap of the Christmas period) were factored into calculations where appropriate. The General Estimating Equation (GEE) analysis was also used where appropriate and is referenced in the text. Graphs were generated using Prism (Version 8.0, GraphPad Software) and the “ggplot2” R package [43].

## 3. Results

### 3.1. Bioactivity Screening and Rice NPN Characteristics

**Figure 1:**
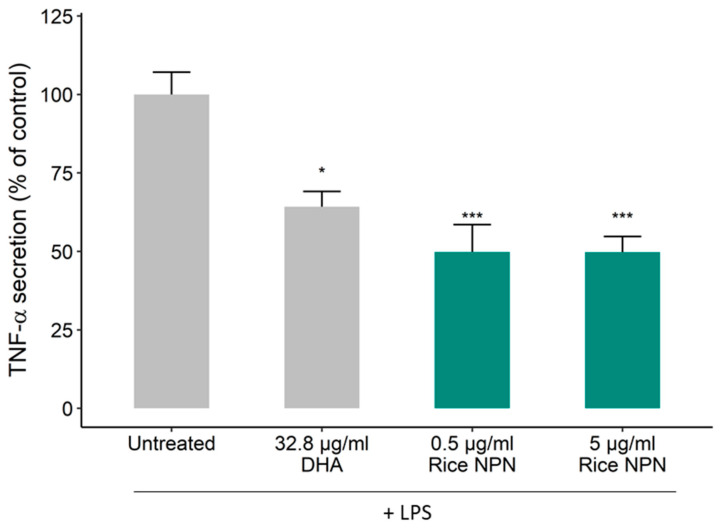
Effect of rice natural peptide network (NPN) on lipopolysaccharides (LPS)-induced TNF-α secretion from differentiated THP-1 macrophages. THP-1 macrophages were treated with rice NPN (0.5 and 5 µg/mL) for 24 h before treating with 100 ng/mL of LPS for 24 h. The secretion of TNF-α was quantified by ELISA. Data presented are the mean ± SEM of at least 3 independent experiments (* *p* < 0.05, *** *p* < 0.001).

**Figure 2:**
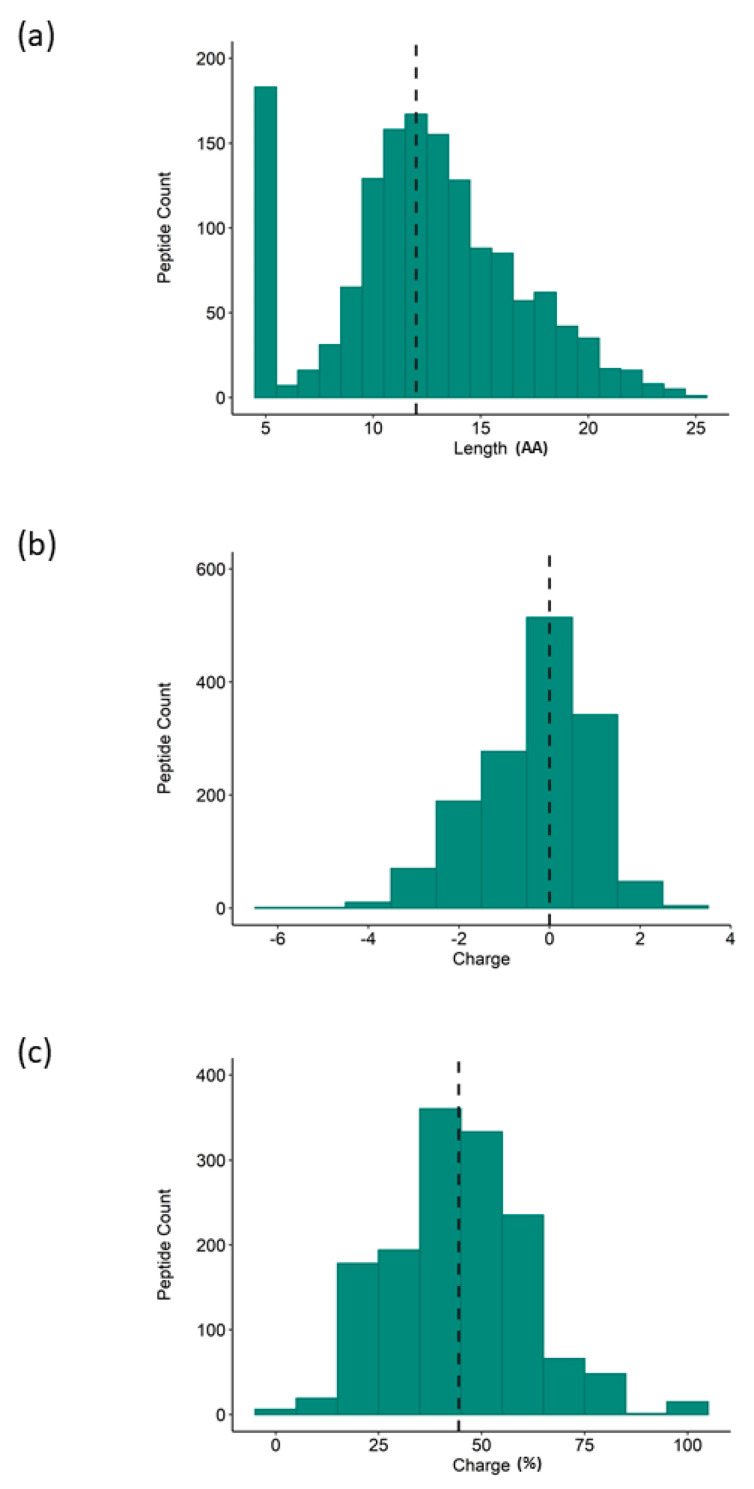
Physicochemical properties of rice NPN peptide profile as determined by LC-MS/MS. Histogram representation of rice NPN peptide distribution according to (**a**) length of peptide sequence, (**b**) charge of constituent peptides, and (**c**) percentage of hydrophobic residues (Hydrophobicity; peptide counts are displayed on the *y* axis; dashed line represents the median.

To explore the latent anti-inflammatory bioactivity within the rice proteome, a hydrolysate was generated and screened for bioactivity. The anti-inflammatory properties of the rice NPN were assessed in vitro in the human THP-1 macrophage cell line. Specifically, differentiated THP-1 macrophages were treated with increasing concentrations of rice NPN for 24 h, before an inflammatory response was induced by further treatment of cells with the bacterial endotoxin LPS. In this model, the concentration of TNF-α secretion was used to quantify the inflammatory response. In Figure 1 we show that, at both test concentrations, treatment with rice NPN resulted in a statistically highly significant (*p* < 0.001) reduction in TNF-α secretion compared to the untreated control. While a dose response was not attained by increasing the concentration of rice NPN, we do observe equivalent efficacy (50% reduction versus untreated) at both 0.5 and 5 µg/mL, which may be indicative of a saturation effect (Figure 2a), importantly, rice NPN did not affect cell viability (up to 500 µg/mL; Figure S1). The rice NPN performed better than a known natural anti-inflammatory ingredient that was used as a positive control and comparator, namely DHA [39]. In Figure 2, we present the physicochemical characteristics of our rice NPN. Here, we see that in terms of peptide length the highest individual frequency observed for the rice NPN is 5 amino acids (AA), however, cumulatively most rice NPN peptides fall within the 10–15 amino acid size range (Figure 2a). Overall, rice NPN peptides feature a global charge range from −4 to 2, where a net neutral charge (0) occurs with the highest frequency (Figure 2b). Finally, in Figure 2c, we see that the majority of peptides within the rice NPN are comprised of approximately 20%–40% hydrophobic residues.

### 3.2. Effects of Constituent Peptides Predicted with Anti-Inflammatory Effects on TNF-α Secretion

**Figure 3:**
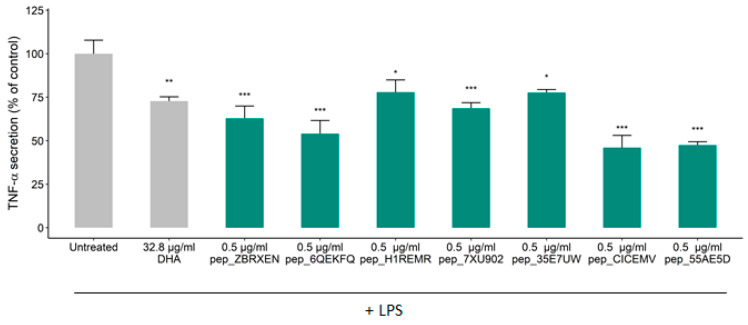
Effects of rice NPN constituent peptides on TNF-α secretion from differentiated THP-1 macrophages. Effect of treatment on the secretion of TNF-α in THP-1 differentiated macrophages after treatment with LPS. THP-1 macrophages were treated with predicted peptides (0.5 µg/mL), or media alone (Untreated) for 24 h before incubation with 100 ng/mL of LPS for 24 h. The secretion of TNF-α was quantified by ELISA. Data presented are the mean ± SEM of at least 3 independent experiments (* *p* < 0.05, ** *p* < 0.01, *** *p* < 0.001).

To further characterize the rice NPN, a set of eight AI-predicted peptides were synthesized for further investigation based on the AI algorithms described in Rein et al., (2019). Sequence information and physicochemical properties of positively predicted peptides are displayed in Table 1. The presence of our selected in vitro predicted peptides in rice NPN was verified by liquid chromatography tandem mass spectrometry (LC-MS/MS) (Figure S2). Here, we see that positively predicted peptides are distributed across five parent rice proteins. Notably, peptide pep_7XU902 is a subsequence of peptide pep_55AE5D. Again, using differentiated THP-1 macrophages, the anti-inflammatory effect of the individual peptides against LPS induced TNF-α was determined. Following a primary screen, we found that seven peptides tested induced a significant reduction in TNF-α secretion compared to untreated cells while one peptide did not (Figure S3), with the effect of five peptides emerging as highly significant (*p* < 0.001; see Figure 3). Similar efficacy to the rice NPN was observed for two of the constituent peptides, where pep_CICEMV and pep_H1REMR respectively produced a 53% and 52% reduction in TNF-α secretion (Figure 3). Further, we found that in all cases the constituent rice NPN peptides selected for testing exhibited more than a 20% reduction in TNF-α secretion (Figure 3).

### 3.3. Supplementation with Rice NPN in an Elderly Cohort Had Beneficial Effects on Physical Activity and Correlated with Markers of Age Associated Inflammation

An elderly cohort in otherwise good health was recruited and ingested rice NPN daily over a 12-week period in a double blinded, placebo-controlled trial (Table S1). The primary endpoint for this study was gut discomfort, as measured by a subjective questionnaire, and while no significant changes were observed on that metric following treatment (Table 2), beneficial effects on serum concentrations of TNF-α and HDL were noted, as well as a beneficial effect on glucose uptake. These were associated with increased performance in functional tests such as a chair stand test and short physical performance battery. No serious adverse events reported (Table S1), as such, rice NPN was considered safe to ingest.

**Figure 4:**
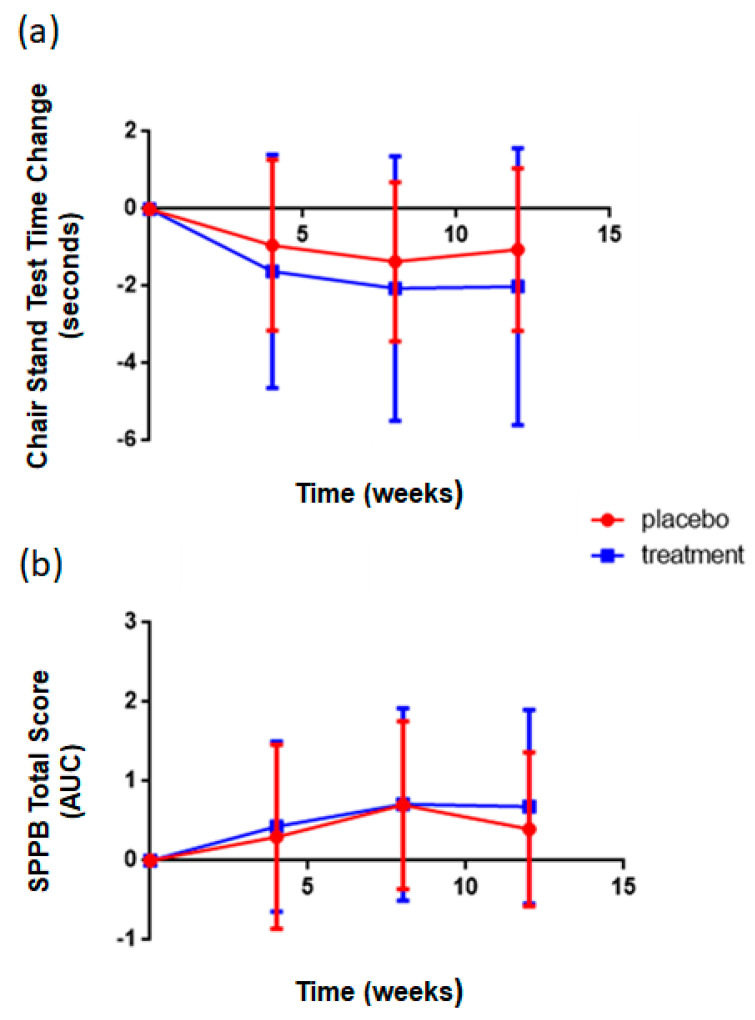
Effect of rice NPN supplementation on lower body strength and overall performance in short physical battery compared to placebo treated controls. (**a**) Changes in repeated chair stand test time at various intervals after the start of consumption of either placebo or rice NPN (treatment) are expressed. Each symbol represents the mean + 1 x +/− s.d. of 10 persons in placebo and at least 27 in rice NPN group. (**b**) Changes in SPPB total score at various intervals after the start of consumption of either placebo or rice NPN are expressed as area under the curve (AUC). Each symbol represents the mean + 1 x +/− s.d. of 10 persons in placebo and at least 28 in rice NPN group. Values are mean ± SEM.

In order to assess the effect of supplementation with the rice NPN, upper and lower body strength were measured by the hand grip test and chair stand tests respectively, at 0, 4, 8, and 12 weeks, while a short physical performance battery was performed at weeks 0 and 12. Performance of the treatment group (rice NPN) in the repeated chair stand test was significantly (*p* = 0.02) improved compared to that of the placebo group, with participants in the treatment group consistently completing chair stands in less time than those in the placebo group (Figure 4a). Two key confounding factors in this dataset were compliance and the time at which the study was measured, i.e., the end of the trial period overlapped with the Christmas period. As such, compliance was possibly not as strict during this period. However, the effect was still observed. When a subgroup analysis was performed, the change observed in the chair test performance was associated with start value at baseline (*p* < 0.001), the higher the start value the larger the overall decrease in repeated chair test time at later intervals in both groups (*p* < 0.001). It was also observed that age inversely correlated with decrease, the older the subject, the lower the decrease (*p* < 0.001). In the Short Physical Performance Battery Test, a significant difference was reported between the physical performance of the treatment group compared to the placebo (*p* = 0.04), with higher scores attained for the treatment group (Figure 4b). A subgroup analysis showed that age (*p* < 0.001); the older the subject, the lower the increase; compliance (*p* < 0.007); the higher the compliance the better the effect and start value at baseline (*p* < 0.001); the higher the value at baseline, the smaller the increase, were all important factors for future trial design. No difference in hand grip test values between placebo and treatment were noted at any time point. Muscle mass measured by Dexa scan showed no significant differences between treatment groups (data not shown).

**Figure 5:**
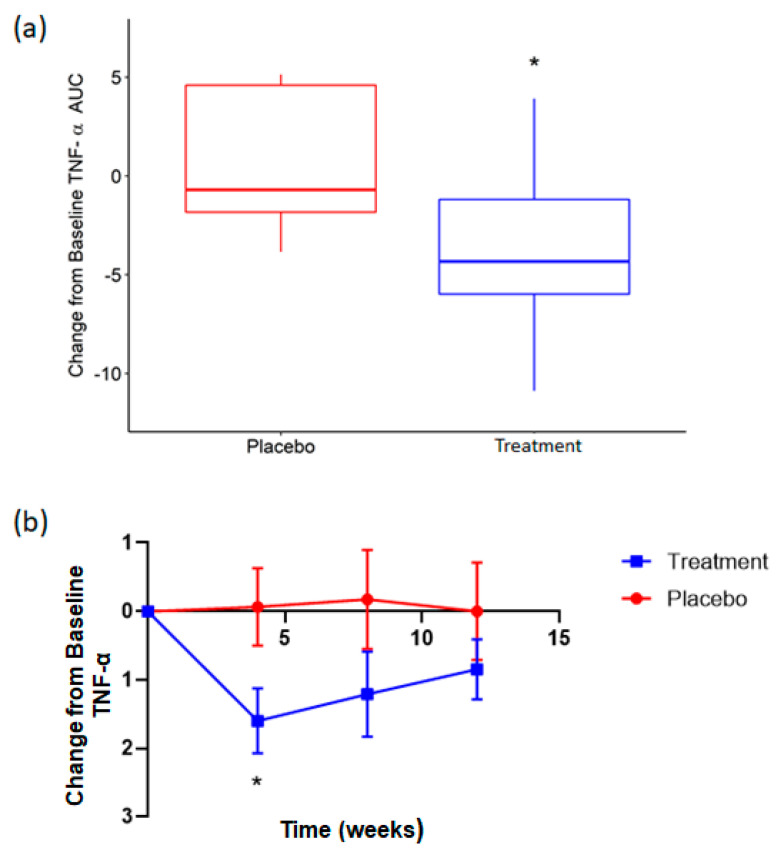
Effect of rice NPN supplementation on circulating concentrations of TNF-α in serum of elderly subjects. Delta area under the curve (**a**) and change from baseline values (**b**) for circulating TNF-α (pg/mL) during in participants consuming placebo (grey circle) or rice NPN (blue circle) for 12 weeks. Values are mean ± SEM (* *p* < 0.05).

Concentrations of circulating cytokines (TNF-α, IL-1β and IL-6) and the inflammatory marker CRP were analyzed across 12 weeks in both cohorts. Total area under the curve (AUC) was used to compare the effect of rice NPN supplementation on these markers over the entire study period. AUC for TNF-α compared to baseline showed a significantly greater reduction (*p* = 0.03) for rice NPN compared to placebo (Figure 5a). When each study time point was subsequently assessed, at week 4 rice NPN was found to display a greater decrease (*p* = 0.04) in circulating TNF-α from baseline than placebo (Figure 5b). While the overall decrease from baseline for TNF-α continued to be greater for rice NPN compared to placebo at week 8 and week 12, this was not significant and diminished with each consecutive time point (Figure 5b). AUC change from baseline for IL-1β, IL-6 and CRP showed no significant differences between treatment groups (see Figure S4a–c, respectively).

### 3.4. Supplementation with Rice NPN Increased Glucose Uptake When Challenged with a Glucose Tolerance Test

**Figure 6:**
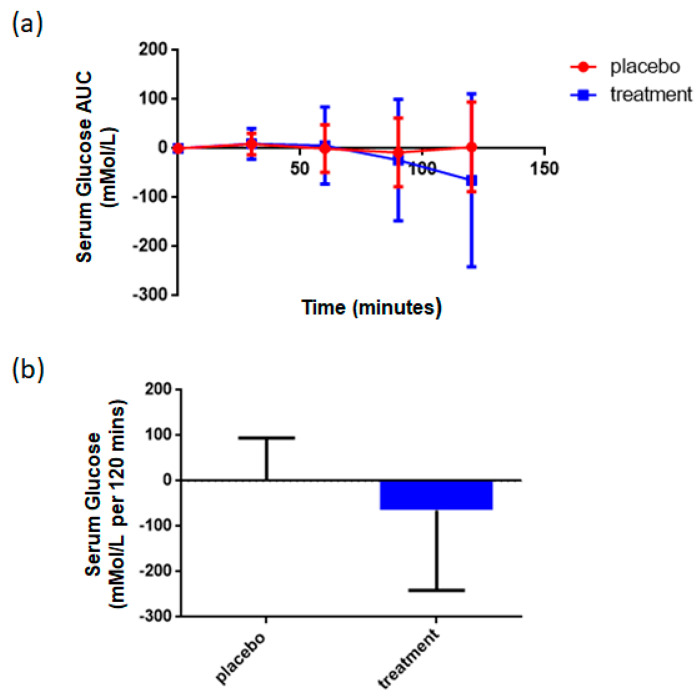
Effect of rice NPN supplementation on serum concentrations of glucose. (**a**) The total area under the curve (AUC) for serum glucose (mmol/l) at various intervals after the start of consumption of either placebo or rice NPN (treatment), starting with “0” at *t* = 0. Each point represents the mean value + 1 x +/− standard deviation of at least 9 observations in placebo and at least 24 in rice NPN group. (**b**) Change in total area under the curve (per 120 min) with respect to the difference in outcome between visits 2 and 5. No outliers removed. Each symbol represents the mean + 1 x +/− standard deviation of 9 persons (in placebo) or 24 persons (in rice NPN group).

Oral glucose tolerance testwas performed as described in Methods, with AUC determined as the key readout. For both placebo and treatment cohorts, the various AUCs were summed. Due to a small number of values not recorded at 120 min, the summation at this time point is based on fewer subjects than earlier time points. Generalized estimation equation (GEE) analysis showed that the reduction during the whole test interval (0–120 min) with respect to the difference in AUC at baseline and week 12 was significant (*p* < 0.001), indicating a positive effect of rice NPN on glucose uptake (Figure 6).

### 3.5. Rice NPN Supplementation Altered LDL and HDL Serum Concentrations

**Figure 7:**
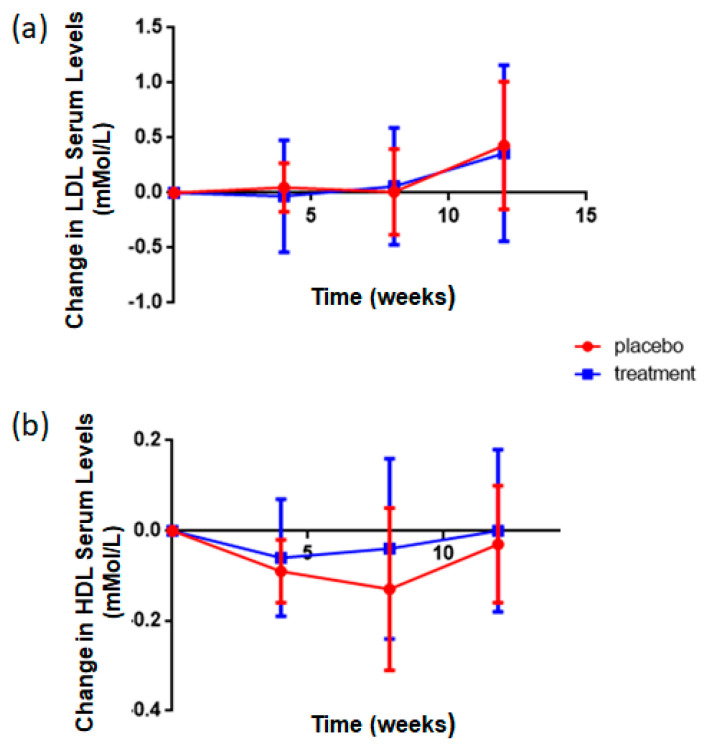
Effect of rice NPN Supplementation on LDL and HDL Serum Concentrations. (**a**) Change in the serum levels of low-density lipoprotein (LDL; mmol/l) in placebo and rice NPN (treatment). Outliers have not been removed. Each symbol represents the mean + 1 x +/− s.d. of 10 persons in placebo and at least 27 in rice NPN group. (**b**) Change in the serum levels of high-density lipoprotein (HDL; mmol/l) in placebo and rice NPN. One outlier has been removed. Each symbol represents the mean + 1 x +/− s.d. of 10 persons in placebo and at least 27 in rice NPN group.

Cholesterol is transported through the blood on proteins called lipoproteins. Two types of lipoproteins carry cholesterol throughout the body: LDL (low-density lipoprotein), which makes up most of the body’s cholesterol—high levels of LDL cholesterol raise your risk for heart disease and stroke; and HDL (high-density lipoprotein) whose function it is to absorb cholesterol and carry it back to the liver [44]. The liver then flushes it from the body. High levels of HDL cholesterol can lower your risk for heart disease and stroke. As shown in Figure 7, relative to placebo supplemented subjects, there was a decrease in LDL and a concomitant increase in HDL concentrations following rice NPN supplementation. The change in HDL values was found to be associated with compliance (*p* < 0.001), the higher the level of compliance, the larger the decrease in LDL serum concentration, and interaction (*p* < 0.007), the time dependent increase in serum concentration values of HDL is larger in rice NPN supplemented group than in placebo.

## 4. Discussion

Food and plant-derived bioactive peptides are increasingly recognized as important preventative immunomodulatory strategies [27,28]; however, to date, there has been limited clinical data to support their role in immune health [45,46] and associated physical health. AI and machine learning approaches offer an opportunity to decode the complexity of these natural sources and identify well characterized and efficacious functional foods [26,31,47,48,49]. To identify a preventative supplement, we used AI to predict an NPN from rice that could decrease TNF-α production. TNF-α was chosen due to its central role in a broad range of inflammatory conditions, and as its concentration increases with age, we planned to target an “inflammaging” population, i.e., an otherwise healthy aged population.

The rice NPN was manufactured and assayed in vitro using differentiated THP1 macrophages stimulated with LPS. We showed that rice NPN could inhibit TNF-α secretion in a dose-dependent manner, with no effect on cell viability, indicating the effect was not due to cell viability issues. We further characterized the NPN to identify the bioactive component peptides contained therein. Their presence was confirmed using MS and activity was confirmed using LPS stimulated THP1 cells.

Rice NPN was then administered to aged volunteers (NCT04450979). Plasma cytokines, glucose tolerance and changes in LDL/HDL concentrations were determined as well as changes in physical performance tests. Administration of rice NPN to an inflammaging population lead to significantly decreased plasma concentrations of TNF-α as well as significant benefits on glucose tolerance and changes in LDL and HDL cholesterol concentrations. These benefits correlated with increased performance in a standard physical battery test and gains in lower limb strength, as measured by a significant improvement in a chair stand test.

Inflammaging is a process whereby our immune system changes with age, rather than a disease. However, as we age, many diseases can develop, and this is thought to be due to this changing immune system and the associated state of low-grade chronic inflammation. With this in mind, we conducted a pilot human study in an “inflammaging” population, i.e., elderly but otherwise healthy. The primary endpoint of this study was to assess digestive discomfort, measured using a subjective self-reporting mechanism. As such, this part of the trial did not meet significance. While it is disappointing that the primary endpoint was not achieved, it is unsurprising given the subjective nature of the readout. However, analysis of objectively measured secondary readouts showed clear benefits on the ability to carry out physical challenges that correlated with a decrease in serum circulating TNF-α, as well as other health benefits such as improved glucose control, a decrease in serum LDL concentration and an increase in HDL concentration. We observed a significant association between the change in TNF-α and the absolute start values of TNF-α at visit 2 (*p* < 0.001), the higher the start value the larger the decrease in TNF-α. This possibly indicated that rice NPN was effective when cytokine concentrations were elevated and not as effective when circulating TNF-α concentrations were low. For example, 44% of subjects that initially displayed serum concentrations higher than 10 pg/mL showed concentrations lower than 10 pg/mL after four weeks of supplementation. This was compared to no effect observed in the placebo. The significant difference between treatment and control observed at week four decreased in intensity at week 8. This suggested that the rice NPN dose used was high and could be significantly lowered to decrease circulating TNF-α in a more transient way over a longer period of time under the current regimen or that the current dose could be maintained but administered less frequently, these observations will be factored in for any future trial design, such as reduced dosage (5 g/day) and intermittent dosing.

Mobility has been shown to be a key determinant of health and quality of life among the elderly [50,51,52,53] and inflammaging has been shown to significantly accelerate morbidity and mortality [54]. Lack of physical activity has been shown to decrease cognitive function, reduce independence, and increase the risk of fractures, falls, and death [55,56,57,58,59,60,61]. The consumption of rice NPN for 12 weeks without additional exercise or other specific dietary alterations resulted in the improvement of lower body muscle strength and significant improvement in mobility performance as measured by the clinically relevant chair stand and SPPB. SPPB has been used as a predictive tool for disability, nursing home admission, and a gradient of risk for mortality [41]. The changes observed were not due to any increased protein intake from the rice NPN as the muscle mass was not significantly different between placebo and rice NPN group. Subjects also carried out their normal day to day activities without additional exercise, indicating the effect is due to supplementation with NPN alone. There was no clear effect on the hand grip test taken to measure upper body strength, but this test has been shown to be a less sensitive measurement for testing intervention compared to the lower limb tests [34].

To the best of our knowledge, this represents the first time that a functional protein ingredient demonstrated inhibitory effects against TNF-α in a placebo-controlled setting. We note that the maximal difference over placebo for rice NPN was seen at week 4, where the greatest effect is attained. While the effect of rice NPN on TNF-α is seen to be moderate post-week 4, this trend was in keeping with those of previous studies [62,63] and may be explored in future work through optimization of study parameters such as population size, dose, or duration. It is also worth noting that the Christmas holidays overlapped significantly with this period of the trial and it is likely subjects did not comply with the study protocol as rigorously during this time. It was statistically shown above that a serious confounding factor affecting efficacy was compliance; the higher the reported level of compliance, the greater the effect observed.

In accordance with our findings here, Rein et al., (2019), investigated a commercial scale up of rice NPN, and also demonstrated a significant reduction in TNF-α in a 24-h kinetic study in healthy subjects [26]. Finally, in line with other studies [64,65,66], despite a positive impact on TNF-α, here we report no observable increase over the test period on other inflammatory markers (IL-6, IL-β and CRP) and, as such, no measurable effect of rice NPN.

We further characterized the rice NPN by identifying seven peptides that exhibited anti-inflammatory efficacy against the LPS-induced TNF-α secretion, with some exhibiting comparable effects to the parent rice NPN. We do note that the reported effects of a number of our peptides are more modest versus those of the rice NPN. However, this is to be expected since parent protein ingredients benefit from the synergistic effects of their constituent peptides [67]. Additionally, the bioavailability of bioactive peptides in rice NPN are under further investigation. While the precise mechanism of action of our peptides against TNF-α is not known, it has been shown previously that both endogenous [68] and exogenous [69] peptides can inhibit signal transduction pathways involved in the expression of inflammatory cytokines [20]. Moreover, some of the peptides presented here have been shown (both in silico and in vitro) to bind with high affinity to TNF-α directly [26] potentially causing the disassembly of this cytokine [70], or preventing the binding of TNF-α to its receptors on human tissues [71,72]. Further work will be required to elucidate which of these potential mechanisms alone or in combination may be responsible for the observed efficacy of these peptides, and indeed the rice NPN.

## 5. Conclusions

Ultimately, in this study we have validated a functional ingredient possessing anti-inflammatory effects in vitro and in human, with rice NPN conferring beneficial effects on physical performance, circulating cytokines, cholesterol, and glucose control in a relevant human population. We have also validated the use of an AI approach for predicting constituent bioactive peptides within a functional ingredient. By adopting this innovative framework, we show that targeted, characterized, functional ingredients offer potential in the maintenance of health or the prevention and treatment of various conditions.

## Figures and Tables

**Table 1:** Sequence and parent protein information for seven positively predicted peptides in rice NPN.

ID	Sequence	Length	Molecular Weight	Charge	Uniprot ID
pep_ZBRXEN	TVFDGVLRPGQL	12	1301.49	0	P14323
pep_6QEKFQ	FYNEGDAPVVAL	12	1294.37	−2	Q0E261
pep_H1REMR	IYGPDTGVDYKDNQMR	16	1871.97	−1	Q0DEV5
pep_7XU902	GYYGEQQQQPGMTR	14	1642.75	0	P29835
pep_35E7UW	IDGYDTPVEGR	11	1221.27	−2	Q0DEV5
pep_CICEMV	NGVLRPGQL	9	953.1	1	P14614
pep_55AE5D	SEEGYYGEQQQQPGMTR	17	1988.05	−2	P29835

**Table 2:** Summary of main readouts from Trial NCT04450979 and associated significance of effects measured.

Readout	Measured By	Effect	*p* Value
Gut Discomfort	Questionnaire	None	NS
TNF-α	ELISA	↓	0.03 *
HDL	Blood Chem Panel	↑	<0.001 **
LDL	Blood Chem Panel	↓	<0.001 **
Oral Glucose Tolerance Test	Glucometer	↑	<0.001 ^#^
Chair Stand	Physical Test	↑	0.02
Hand Grip	Physical Test	None	NS
SPPB	Physical Test	↑	0.04

* Overall area under the curve (AUC) across the entire study reported; ** Values were associated with compliance; ^#^ GEE analysis showed that the reduction during the whole test interval (0–120 min) with respect to the difference in AUC at baseline and week 12 was significant (*p* < 0.001).

## Supplementary Materials

The following are available online at https://www.mdpi.com/2304-8158/9/9/1147/s1, Figure S1: Cell viability following rice NPN treatment Figure S2: Ion spectra for predicted peptides detected in rice NPN. Figure S3: Predicted peptide did not reduce LPS induced TNF-α secretion. Table S1: Subject Anthropometric Data. Figure S4: AUC change from baseline for (a) IL-1β, (b) IL-6 and (c) C-Reactive Protein.
