# Supplementary material for: An Artificial Intelligence Characterised Functional Ingredient, Derived from Rice, Inhibits TNF-α and Significantly Improves Physical Strength in an Inflammaging Population
Source: Foods. 2020 Aug 20;9(9):1147. doi: 10.3390/foods9091147 (PMC7555431; doi:10.3390/foods9091147)
Supplement: Supplementary file 1 [file foods-09-01147-s001.pdf]

**An AI characterised functional ingredient derived from rice inhibits TNF-α and significantly improves physical strength in an inflammaging population**

Kathy Kennedy[1], Brian Keogh[1], Cyril Lopez[1], Alessandro Adelfio[1], Brendan Molloy[1], Alish Kerr[1], Audrey M. Wall*[1], Gaël Jalowicki[1], Thérèse A. Holton[1] and Nora Khaldi[1]

[1] Nuritas Ltd, Joshua Dawson House,
Dawson St, Dublin 2, D02 RY95, Ireland.

Supplementary Materials

***Corresponding Author**

Audrey Wall

Email: wall.audrey@nuritas.com

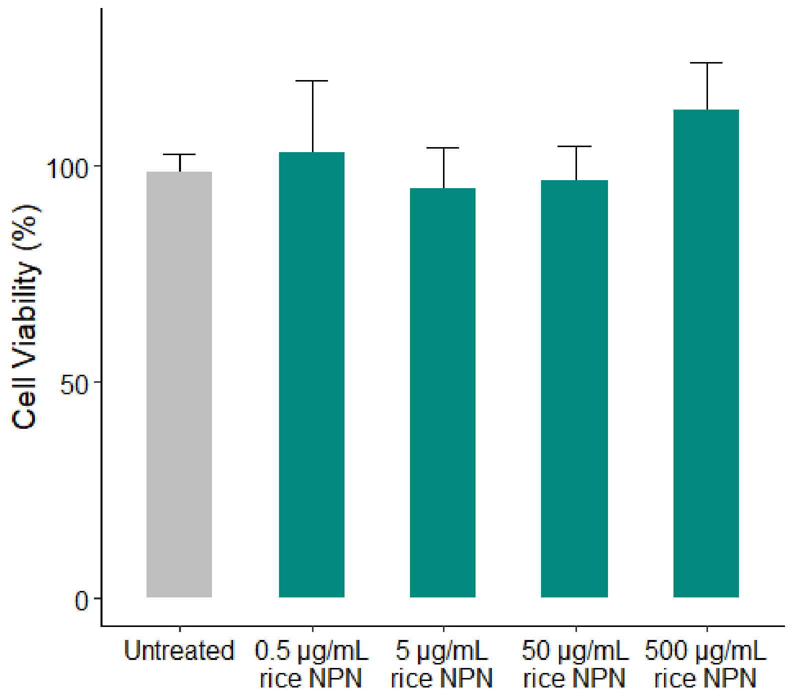


**Figure S1. Cell viability following rice NPN treatment.** Percentage viability of Thp-1 cells with increasing concentrations of rice NPN. Data presented are the mean ± SEM of at least 2 individual experiments carried out in at least triplicate.

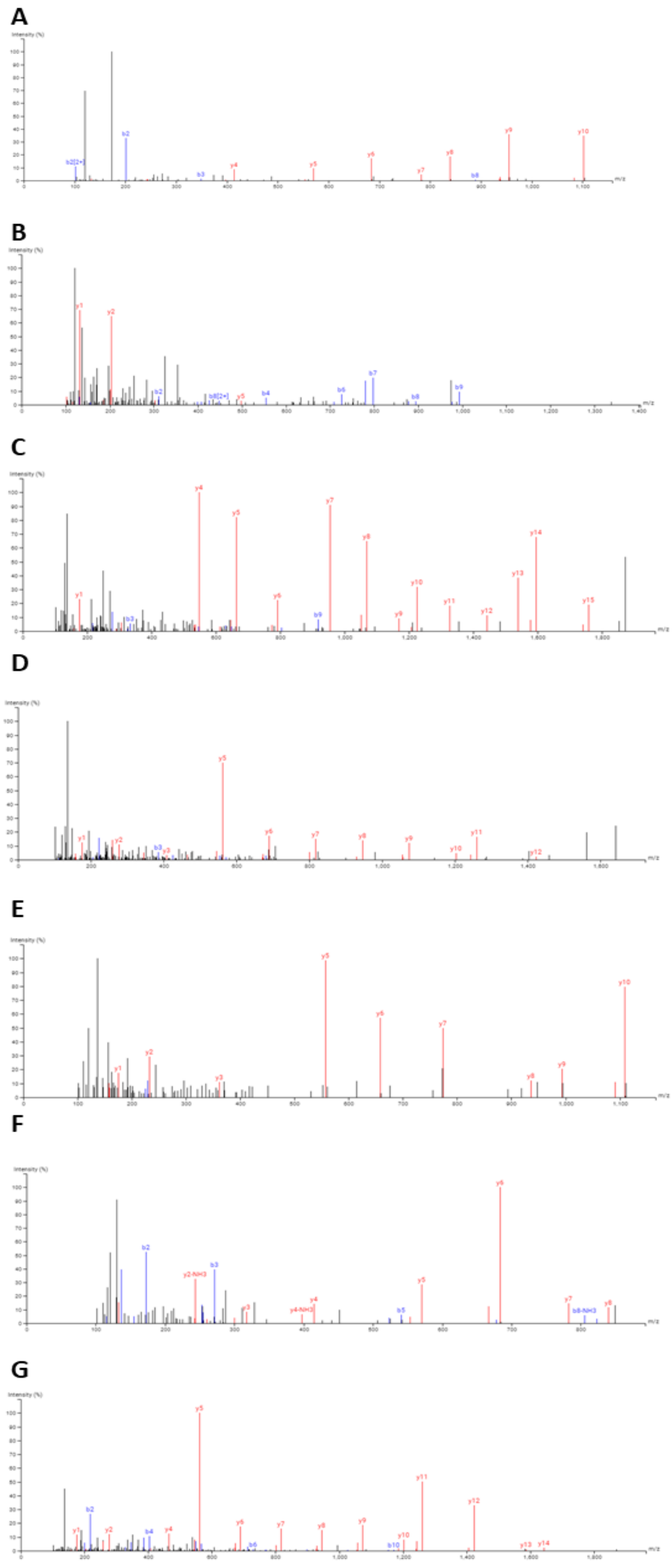


**Figure S2: Ion spectra for predicted peptides detected in rice NPN.** Ion spectra are shown for peptides (a) pep_ZBRXEN (b) pep_6QEKFQ (c) pep_H1REMR (d) pep_7XU902 (e) pep_35E7UW (f) pep_CICEMV and (g) pep_55AEE5D.

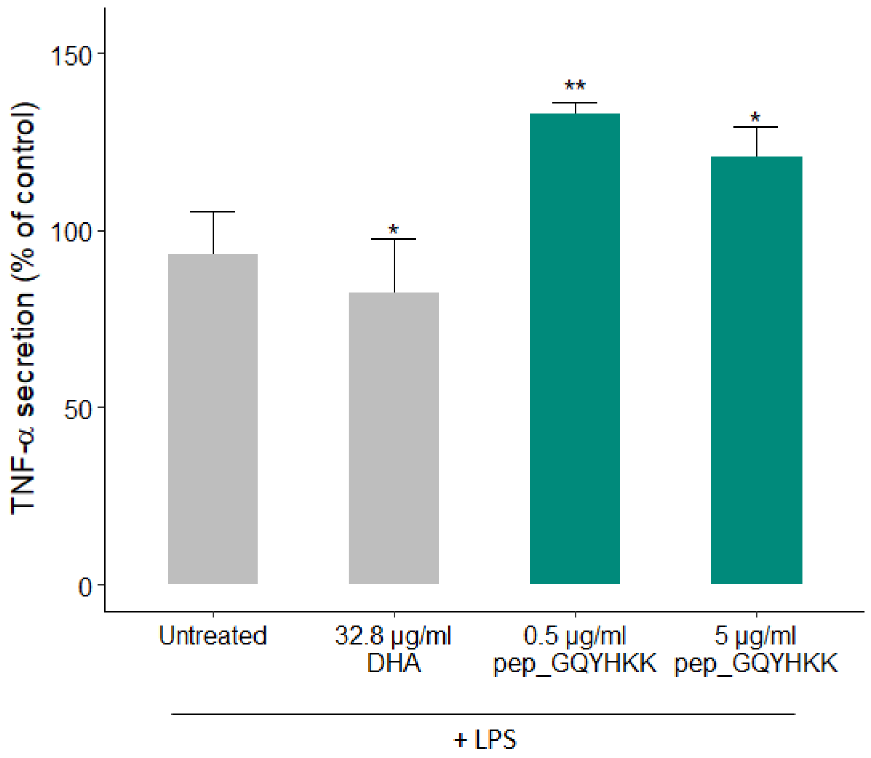


**Figure S3: Predicted peptide did not reduce LPS induced TNF-α secretion.** Treatment with constituent peptide, pep_GQYHKK, did not reduce the secretion of TNF-α in THP-1 differentiated macrophages after treatment with LPS. THP-1 macrophages were treated with predicted peptide (0.5 - 5 µg/mL) for 24 hours before treating with 100 ng/mL of LPS for 24 hours. The secretion of TNF-α was quantified by ELISA. Data presented are the mean ± SEM of 1 independent experiment in triplicate (*P <0.05 **P <0.0

**Table S1** Subject Anthropometric Data

| | All | Male | Female |
|---|---|---|---|
| Gender n (%) | 40 (100%) | 15 (37.5%) | 25 (62.5%) |
| Age (years)<br>(Mean ± S.E.) | 68.49 ± 0.51 | 69.34 ± 0.86 | 67.99 ± 0.62 |
| Weight (Kg)<br>(Mean ± S.E.) | 74.42 ± 1.71 | 80.74 ± 2.73 | 71.2 ± 1.79 |
| Height (m)<br>(Mean ± S.E.) | 1.67 ± 0.01 | 1.73 ± 0.01 | 1.63 ± 0.01 |
| BMI ($Kg/m^2$)<br>(Mean ± S.E.) | 26.65 ± 0.42 | 26.87 ± 0.67 | 26.52 ± 0.56 |
| Respiratory Rate<br>(Mean ± S.E.) | 16.85 ± 0.31 | 17.2 ± 0.48 | 16.63 ± 0.4 |
| Blood pressure<br>(Mean ± S.E.) | | | |
| Diastolic | 80.48 ± 1.56 | 81.8 ± 3.2 | 79.68 ± 1.65 |
| Systolic | 131.0 ± 2.52 | 132.6 ± 4.05 | 130.04 ± 3.27 |
| | | Treatment | Placebo |
| **Adverse events** | Nausea and loose stool | 3 | 0 |
| | Indigestion | 1 | 1 |
| | Constipation | 1 | 0 |
| | Rash / Joint Pain | 0 | 1 |
| **Serious Adverse Events** | | None | None |

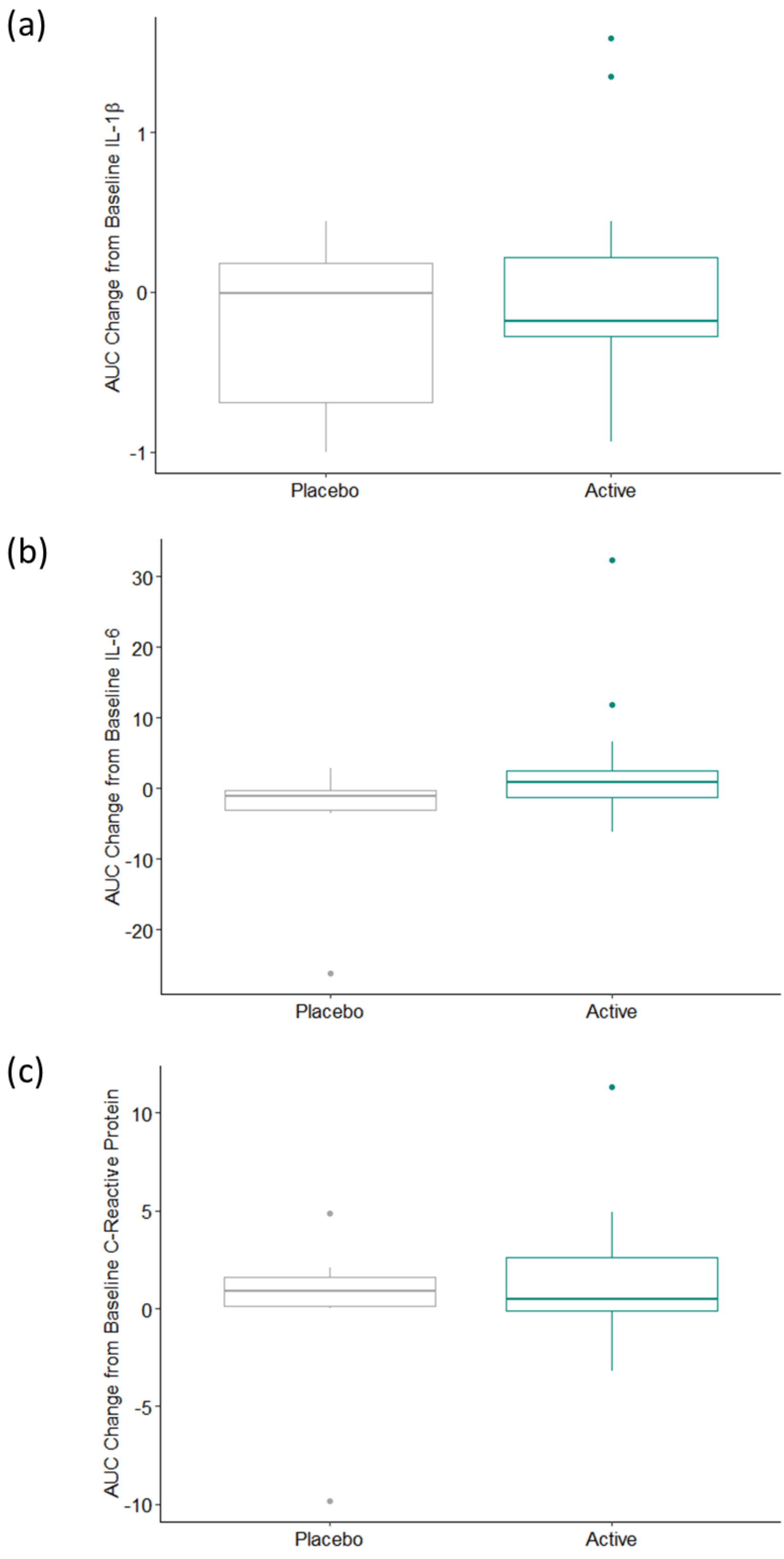


**Figure S4:** AUC change from baseline for (a) IL-1β, (b) IL-6 and (c) C-Reactive Protein.
